# Carvacrol Alleviates Ischemia Reperfusion Injury by Regulating the PI3K-Akt Pathway in Rats

**DOI:** 10.1371/journal.pone.0104043

**Published:** 2014-08-01

**Authors:** Lida Suo, Kai Kang, Xun Wang, Yonggang Cao, Haifeng Zhao, Xueying Sun, Liquan Tong, Feng Zhang

**Affiliations:** 1 Department of General Surgery, the Fifth Affiliated Hospital of Harbin Medical University, Daqing, Heilongjiang Province, China; 2 Department of General Surgery, Daqing Oilfield General Hospital, Daqing, Heilongjiang Province, China; 3 Department of Pharmacology, Daqing Campus of Harbin Medical University, Daqing, Heilongjiang Province, China; 4 The Hepatosplenic Surgery Center, Department of General Surgery, the First Affiliated Hospital of Harbin Medical University, Harbin, Heilongjiang Province, China; Duke University Medical Center, United States of America

## Abstract

**Background:**

Liver ischemia reperfusion (I/R) injury is a common pathophysiological process in many clinical settings. Carvacrol, a food additive commonly used in essential oils, has displayed antimicrobials, antitumor and antidepressant-like activities. In the present study, we investigated the protective effects of carvacrol on I/R injury in the Wistar rat livers and an *in vitro* hypoxia/restoration (H/R) model.

**Methods:**

The hepatoportal vein, hepatic arterial and hepatic duct of Wistar rats were isolated and clamped for 30 min, followed by a 2 h reperfusion. Buffalo rat liver (BRL) cells were incubated under hypoxia for 4 h, followed normoxic conditions for 10 h to establish the H/R model *in vitro*. Liver injury was evaluated by measuring serum levels of alanine aminotransferase (ALT) and aspatate aminotransferase (AST), and hepatic levels of superoxide dismutase (SOD), catalase (CAT), glutathione (GSH) and malondiadehyde (MDA), and hepatic histology and TUNEL staining. MTT assay, flow cytometric analysis and Hoechst 33258 staining were used to evaluate the proliferation and apoptosis of BRL cells *in vitro*. Protein expression was examined by Western Blot analysis.

**Results:**

Carvacrol protected against I/R-induced liver damage, evidenced by significantly reducing the serum levels of ALT and AST, histological alterations and apoptosis of liver cells in I/R rats. Carvacrol exhibited anti-oxidative activity in the I/R rats, reflected by significantly reducing the activity of SOD and the content of MDA, and restoring the activity of CAT and the content of GSH, in I/R rats. In the *in vitro* assays, carvacrol restored the viability and inhibited the apoptosis of BRL cells, which were subjected to a mimic I/R injury induced by hypoxia. In the investigation on molecular mechanisms, carvacrol downregulated the expression of Bax and upregulated the expression of Bcl-2, thus inhibited the activation of caspase-3. Carvacrol was also shown to enhance the phosphorylation of Akt.

**Conclusion:**

The results suggest that carvacrol could alleviate I/R-induced liver injury by its anti-oxidative and anti-apoptotic activities, and warrant a further investigation for using carvacrol to protect I/R injury in clinic.

## Introduction

Liver ischemia reperfusion (I/R) injury is a common pathophysiological process in many clinical settings, such as hypovolemic shock, liver tumor resection and liver transplantation [1]. The mechanisms contributing to the pathophysiology of liver I/R injury include reactive oxygen species (ROS), calcium overload, cytokines and cell apoptosis [2]. It was well known that reactive oxygen species (ROS) are related with pathogenesis of I/R injury, and contribute to I/R-induced injury, as they cause direct cellular injury and activate a cascade of mediators leading to microvascular changes, increased apoptosis and acute inflammatory changes with increased necrosis [3] [4]. Superoxide dismutase (SOD) plays an important role in maintaining the oxidative antioxidant balance [5], malondiadehyde (MDA) is the product of free radical lipid peroxidation and its activity represents the capability of subsequently causing changes in a variety of cytokines [6], the capability of glutathione (GSH) and catalase (CAT) reductase are to scavenge free radicals in the I/R injury [7] [8] [9]. Many studies take the MDA as a standard to evaluate severity of liver I/R injury. Under normal physiological conditions, the ROS can be quickly broken down by endogenous antioxidant enzymes and low-molecular weight antioxidants such as SOD, CAT and GSH. Apoptosis is an important mechanism in liver I/R injury. It is reported that 50%–70% liver sinusoidal endothelial cells and 40%–60% hepatocyte shaped apoptosis after I/R in the rat liver [10].

Carvacrol [isopropyl-ortho-cresol, C_6_H_3_(OH)(C_3_H_7_), CAR] is a monoterpenic phenol naturally found in various plants belonging to the family Lamiaceae [11]. As a food additive, it has been widely used in the food industry. It has been reported that CAR has bactericidal, fungicidal and insecticidal activities [12], [13], and anti-tumor activity [14]. The main mechanisms accounting for its activities are its antioxidant properties. Therefore, we hypothesized that CAR may protect livers from I/R injury. In addition, the phosphatidylinositol-3-kinase/protein kinase B (PI3K/Akt) signal transduction pathway is a key cellular signaling pathway involved in regulating responses to I/R injury [15], [16], thus we have also explored whether CAR could affect this pathway.

## Materials and Methods

### Ethics statement

Male Wistar rats weighing 200–240 g were obtained from the Animal Research Center at Harbin Medical University, Harbin, China. All animal procedures were approved the by Experimental Animal Ethic Committee of Harbin Medical University.

### Induction of ischemia reperfusion injury

The IR protocol has been previously reported [17]. Briefly, Wistar rats underwent a median laparotomy under anesthesia induced by chloral hydrate (200 mg/kg). The portal vein, hepatic arterial and hepatic duct were isolated and clamped for 30 min, followed by a 2 h reperfusion. Animals were randomly divided into three groups (n = 8, per group): Sham group: rats underwent laparotomy without clamping blood vessel; CAR group: rats received i.p. injection of CAR (Sigma Chemical Co., USA) at a dose of 75 mg/kg [18]; I/R group: ischemia for 30 min, then following 2 h reperfusion; I/R+CAR group: I/R rats received an i.p. injection of CAR at a dose of 75 mg/kg. At the end of experiments, blood samples were collected, and livers harvested and stored at −80°C.

### Biochemical Analysis

Serum alanine aminotransferase (ALT) and aspartate aminotransferase (AST) were estimated by an automated biochemical analyzer (Toshiba, Tokyo, Japan) [19]. ALT and AST in serum samples were expressed U/L.

### Measurement of SOD, CAT, GSH and MDA

The enzymatic activities of SOD and CAT and levels of GSH and MDA in liver tissues were measured with commercial kits from Nanjing Jian Cheng Bioengineering Institute. The activity of SOD was quantified as the rate inhibition of nucleotide oxidation. The level of CAT was quantified by flaxen complex compound at the wavelength of 405 nm. The contents of GSH were determined by the ratio of the reduced glutathione than the oxidized glutathione. The MDA was estimated by evaluating the thiobarbituricacid (TBA) reacting substances at the wavelength of 532 nm [20]. The results for SOD, CAT, GSH and MDA were defined as U/mg protein, U/mg protein, mg/gram protein and nmol/mg protein, respectively.

### Histopathological Examination

Liver specimens were fixed in 4% paraformaldehyd. Six micrometer thick sections were stained with hematoxylin and eosin (HE) and examined by light microscopy in a double-blind manner. The hepatic damage was evaluated with a histopathological scoring system as described previously [17], [21]. Briefly, the assessment was expressed as the sum of the individual score grades from 0 (no findings), 1 (mild), 2 (moderate), to 3 (severe) for each of the following six parameters: cytoplasmic color fading, vacuolization, nuclear condensation, nuclear fragmentation, nuclear fading and erythrocyte stasis.

### TUNEL staining

Terminal deoxynucleotidyl transferase-mediated dUTP nick end labeling (TUNEL) was performed with an apoptosis detection kit (Roche, Basel, Switzerland). Each field was randomly selected without significant necrosis in 10 high-power fields (×400) for counting TUNEL - positive cells. The index of TUNEL was calculated according to the numbers of the total nuclei and the cells with brown nuclei [22].

### Cell model and MTT-cell proliferation assay

The Buffalo rat liver (BRL) cell line was provided by the Cell Bank of Academy in China (Shanghai, China). The cells were cultured in Dulbecco's modified Eagle's medium (DMEM) containing 10% (v/v) fetal bovine serum (FBS, Sigma Chemical Co., USA), penicillin (100 units/mL), and streptomycin (100 units/mL) at 37°C in a humidified atmosphere containing 5% (v/v) CO_2_. The effect of CAR on the viability of BRL cells was determined by 3-(4, 5-dimethyl-thiazol-2-yl)-2, 5-diphenyltetrazoliumbromide (MTT) assay. BRL cells were seeded in 96 well culture plates at the density of 4×10^3^ cells/well with different concentrations of CAR (0.3, 0.6, 1.2 or 2.4 mM), and incubated in a hypoxia incubator for 4 h, followed by under a normal culture condition for 10 h, to mimic hypoxia/restoration (H/R). The cell culture was removed and 20 µL of MTT was added to each well. The cells were further cultured at 37°C for 4 h, and then the cell culture was removed and 150 µL of DMSO was added to dissolve formazan completely. Absorbance of formazan was determined at 490 nm by the microplate reader and % survival was evaluated. All experiments were repeated in triplicate. According to the results of MTT assay, BRL cells were divided into four groups: (1) Control group; (2) H/R group; (3) H/R+CAR group; (4) H/R+LY (LY294002)+CAR group. BRL cells incubated in cell hypoxia experiment culture box for 4 h followed incubated in cell culture box for 10 h to establish the simulated H/R group while the control group was incubated in the normal culture medium [23]. CAR (0.6 mM) was added into the culture medium at the beginning of H/R to establish the H/R+CAR group. LY294002 (the inhibitor of phosphatidylinositol 3-kinase; 25 µM) [24] was added 2 h before addition of CAR (0.6 mM) at the beginning of H/R to establish the H/R+LY+CAR group.

### Flow cytometry assay

The apoptotic rate of BRL cells was detected by flow cytometry as described [25]. Briefly, the BRL cells were washed three times with PBS, and stained with annexin-V-fluorescein and Puffer (Roche, Nonnenwald, Penzberg, Germany), according to the manufacturer's instructions. Cell fluorescence was determined with a flow cytometer. All experiments were repeated in triplicate.

### Cell morphological assay

Cells were cultured in six well culture plates and treated as described above. Cell morphology was assessed by Hoechst 33258 staining [26]. Briefly, cells were washed with PBS three times for 5 min, and then stained with 1 µL Hoechst 33258 in 1 mL solution in each well and incubated for 30 min in the dark. Stained cells were washed with PBS three times for 5 min, and then observed under a fluorescence microscope by using 350 nm stimulation and 460 nm emission. All experiments were repeated in triplicate.

### Western blot analysis

Liver samples were homogenized and lysed in a buffer containing 10 mM Tris-HCl (pH 8.0), 150 mM NaCl, 10% glycerol, 1% NP-40, 5 mM EDTA and protease inhibitor, and then centrifuged at 13,500×g for 20 min at 4°C. The resulting supernatants were used for Western Blot analysis. BRL cells was collected and lysed with RIPA buffer. Total proteins were evaluated using the BCA protein assay reagent. Protein samples were loaded on a 10% sodium dodecyl sulfate-polyacrylamide gel electrophoresis (SDS-PAGE) and then transferred to PVDF membranes. The membranes were blocked in TBST (137 mM NaCl, 20 mM Tris HCl (pH 7.6), and 0.1% (v/v) Tween 20) containing 5% (w/v) nonfat dry milk at 37°C for 2 h. The membranes were incubated overnight at 4°C with antibodies against Caspase-3 (1∶300), Bax (1∶200), Bcl-2 (1∶200), Akt (1∶500), phosphorylated Akt (Ser473) (1∶500) and β-actin (1∶2000), respectively. All the primary antibodies were obtained from Cell Signaling Technology (MA, USA). After washing with TBST at 10 min intervals, the membranes were incubated with the secondary alkaline phosphatase-IgG (1∶5000; Santa Cruz) for 1 h at room temperature in the dark. The membrane was developed with enhanced chemiluminesecence (ECL; Applygen) and exposed to X-ray, then washed 3 times with PBS and scanned by an Odyssey infrared imaging system (LI-COR, Lincoln, NB) at a wave length of 800 nm. The β-actin was regarded as an internal reference for relative quantification.

### Statistical analysis

Data are expressed as mean values ± standard error of the mean (SEM). Comparisons among multiple groups were made with a one-way analysis of variance (ANOVA) followed by the Dunnett's test. “P<0.05” was used for statistical significance.

## Results

### CAR reduces the serum levels of ALT and AST in I/R rats

**Figure 1:**
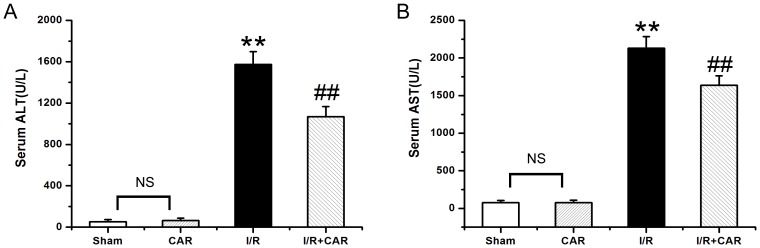
CAR reduces the serum levels of ALT and AST in I/R rats. The serum levels of ALT (A) and AST (B) were measured, and values were expressed as mean ± SEM (n = 8). NS, no significant difference. ^**^ *P*<0.001 compared to the sham group; ^##^ *P*<0.001 compared to the I/R group.

As shown in Fig. 1A, the level of ALT in the I/R group was 1574.12±124.41 U/L, which was significantly higher (*p*<0.001, n = 8) than in the sham group (51.45±21.03 U/L). The ALT level in the I/R+CAR group was 1068.00±98.20 U/L, which was significantly lower (*p*<0.05, n = 8) than in the I/R group. There was no significant difference in ALT between the sham and CAR groups (64.75±21.85 U/L, *p*>0.05, n = 8). Similarly, the level of AST in the I/R group was 2131.15±154.40 U/L, which was significantly higher (*p*<0.001, n = 8) than in the sham group (75.37±31.55 U/L) (Fig. 1B). The AST level in the I/R+CAR group was 1638.70±124.26 U/L, which was significantly lower (*p*<0.05, n = 8) than in the I/R group. There was no difference in AST between the sham and CAR groups (76.85±32.73, *p*>0.05, n = 8) (Fig. 1B).

### CAR exhibits anti-oxidative activity in IR rats

**Figure 2:**
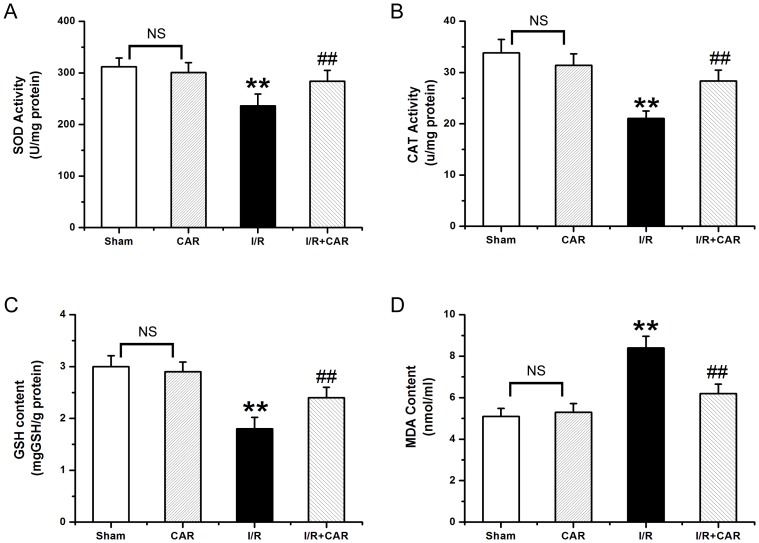
Car exhibits anti-oxidative activity in I/R rats. The hepatic levels of SOD (A), CAT (B), GSH (C) and MDA (D) were measured, and values were expressed as mean ± SEM (n = 8). NS, no significant difference. ^**^ *P*<0.001 compared to the sham group; ^##^ *P*<0.001 compared to the I/R group.

As shown in Fig. 2A, the activity of SOD in the I/R group was 235.76±23.34 U/mg protein, which was significantly lower (*p*<0.05, n = 8) than in the sham group (312.12±17.38 U/mg protein). The activity of SOD in the I/R+CAR group was 284.39±20.81 U/mg protein, which was significantly higher (*p*<0.05, n = 8), than in the I/R group, and no significantly different from the sham group. There was no difference in SOD between the sham group and the CAR group (301.25±18.76, *p*>0.05, n = 8). Similarly, as shown in Fig. 2B, the activity of CAT in the I/R group was 21.06±3.44 U/mg protein, which was significantly lower (*p*<0.05, n = 8) than in the sham group (33.82±4.61 U/mg protein). The activity of CAT in the I/R+CAR group was 28.36±5.10 U/mg protein, which was significantly higher (*p*<0.05, n = 8) than in the I/R group. There was no difference in CAT between the sham group and the CAR group (31.38±4.28, *p*>0.05, n = 8). Furthermore, the content of GSH in the I/R group was 1.84±0.22 µg/g protein, which was significantly lower (*p*<0.05, n = 8) than in the sham group (3.21±0.21 µg/g protein) (Fig. 2C). The content of GSH in the I/R+CAR group (2.36±0.20 µg/g protein) was significantly higher (*p*<0.05, n = 8) than in the I/R group. There was no difference in GSH between the sham group and the CAR group (2.89±0.19, *p*>0.05, n = 8) (Fig. 2C). In addition, the content of MDAin the I/R group (8.43±0.56 nmol/mg protein) was significantly higher (*p*<0.05, n = 8) than in the sham group (5.10±0.38 nmol/mg protein) (Fig. 2D). The content of MDA in the I/R+CAR group (6.21±0.45 nmol/mg protein) was significantly lower (*p*<0.05, n = 8) than in the I/R group, and not significantly different from that in the sham group. There was no significantly difference in MDA between the sham group and the CAR group (5.27±0.42, *p*>0.05, n = 8) (Fig. 2D).

### CAR inhibits apoptosis of liver cells in I/R rats

**Figure 3:**
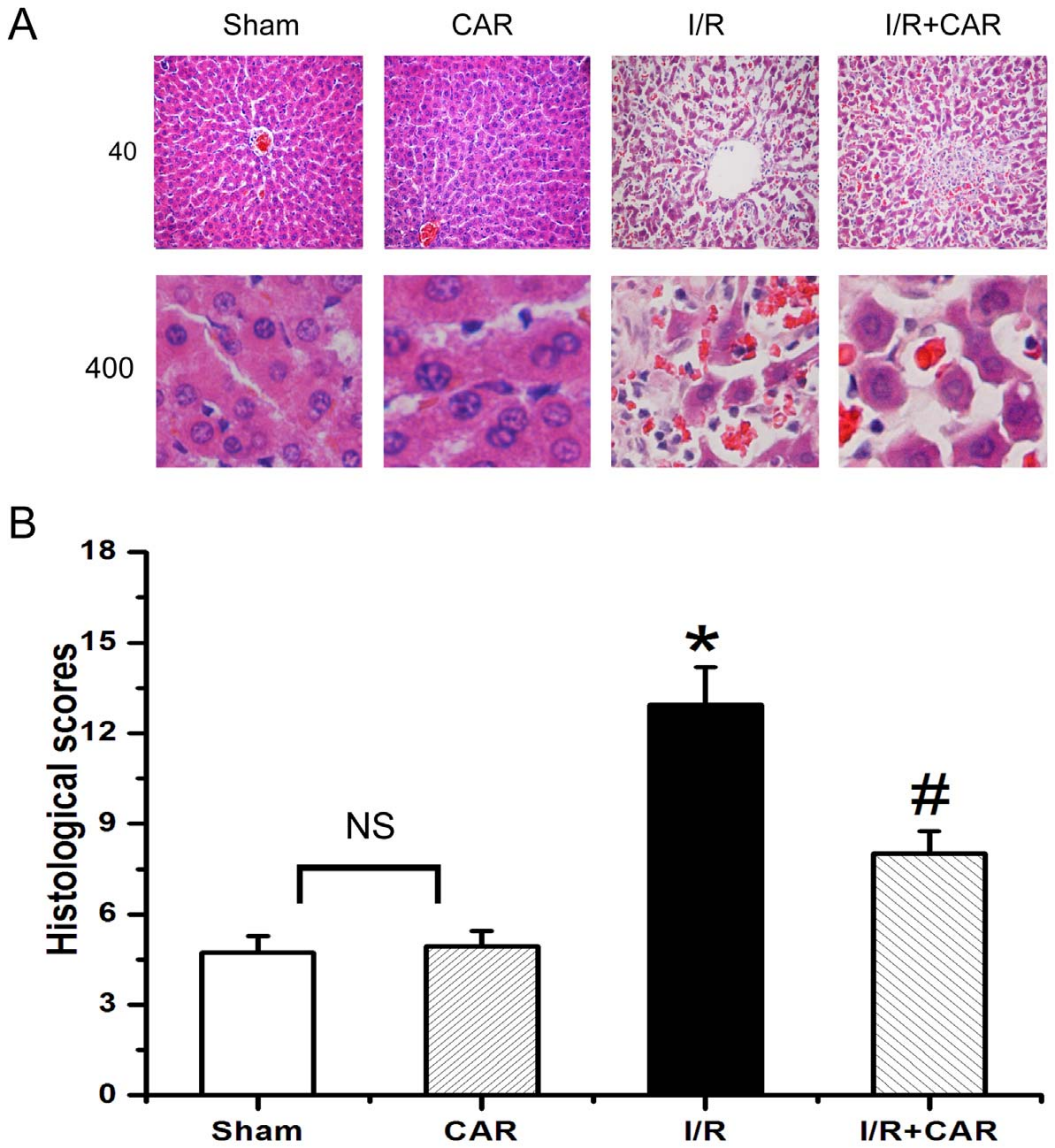
CAR attenuates liver damage in I/R rats. (A) Representative images were taken from HE-stained liver sections (Upper panel: 40× magnification; lower panel: 400× magnification) from the sham, CAR, I/R and I/R+CAR groups. The above liver sections were assessed for histological scores. Values were expressed as mean ± SEM (n = 8). NS, no significant difference. ^*^ *P*<0.05 compared to the sham group; ^#^ *P*<0.05 compared to the I/R group.

**Figure 4:**
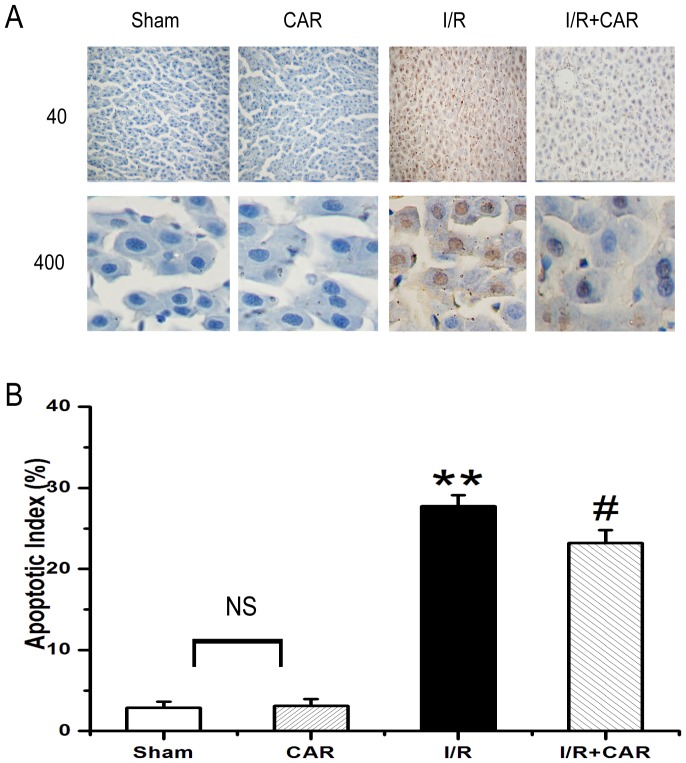
CAR inhibits apoptosis of liver cells in I/R rats. (A) Representative images were taken from TUNEL-stained liver sections (Upper panel: 40× magnification; lower panel: 400× magnification) from the sham, CAR, I/R and I/R+CAR groups. (B) The number of TUNEL-positive cells were counted from the above liver sections. Values were expressed as mean ± SEM (n = 8). NS, no significant difference. ^**^ *P*<0.001 compared to the sham group; ^#^ *P*<0.05 compared to the I/R group.

Karyopyknosis, karyolysis and nuclear fragmentation of hepatocytes were frequently observed in livers from the IR group, while they were scarcely found in livers from the sham group. (Fig. 3A). Treatment of CAR had no effect on hepatic histology, but greatly reduced the number of karyopyknosis, karyolysis and nuclear fragmentation of hepatocytes compared with the I/R group (Fig. 3A). The quantitative analysis of hepatic histopathology further supported the above observation (Fig. 3B). Thus, there was no significant difference in histological scores between the sham and CAR groups; while the histological score in the I/R group was significantly higher (*p*<0.05, n = 8) than in the sham group, and the histological score in the I/R+CAR group was significantly lower (*p*<0.05, n = 8) than in the I/R group. We further examined whether hepatic damage was due to the apoptosis of liver cells. As shown in Fig. 4 A and B, there was few TUNEL-positive cells in rat livers from the sham group, whereas a large number of TUNEL-positive cells were found in the I/R group. There was no significant difference in the number of TUNEL-positive cells in rat livers between the sham and CAR groups. Compared with I/R group, the number of TUNEL-positive cells was significantly decreased in the I/R+CAR group (Fig. 4).

### CAR restores the viability of I/R BRL cells *in vitro*

**Figure 5:**
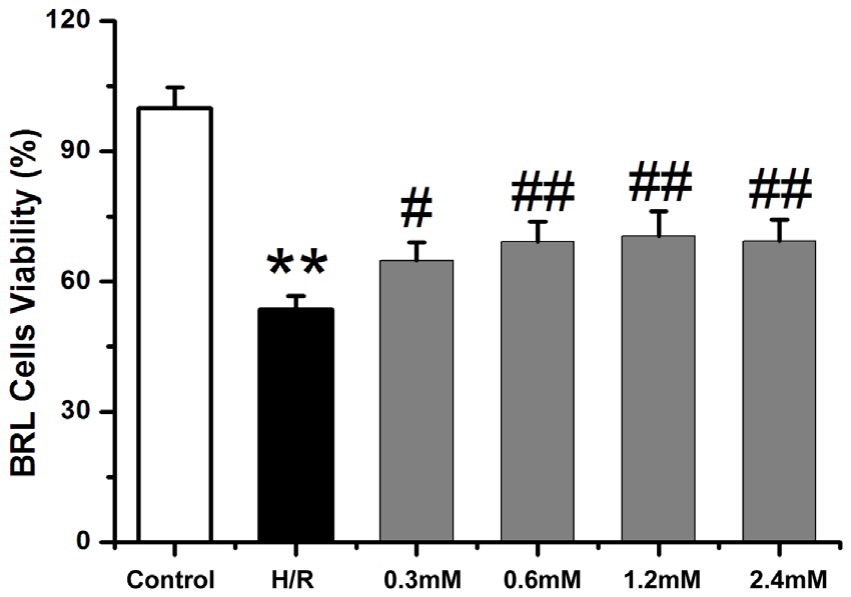
CAR restores on the viability of H/R BRL cells *in vitro*. Untreated BRL cells (control), BRL cells subjected to H/R hypoxia, and BRL cells subjected to H/R hypoxia and treated with different concentrations of CAR (0.3, 0.6, 1.2 or 2.4 mM) were analyzed for the viability. Values were expressed as mean ± SEM (n = 8). ^**^ *P*<0.001 compared to the control group; ^#^ *P*<0.05 and ^#^ *P*<0.001 compared to the H/R group.

BRL cells had a significant lower viability when exposed to H/R compared with untreated controls. However, when the H/R cells were treated with different concentration (0.3, 0.6, 1.2, 2.4 mM) of CAR, the viability of cells was significantly increased compared with H/R cells without CAR treatment. The results indicate that CAR could increase the viability of H/R BRL cells at the tested range of concentrations (Fig. 5).

### CAR inhibits apoptosis of I/R BRL cells via the PI3K pathway *in vitro*

**Figure 6:**
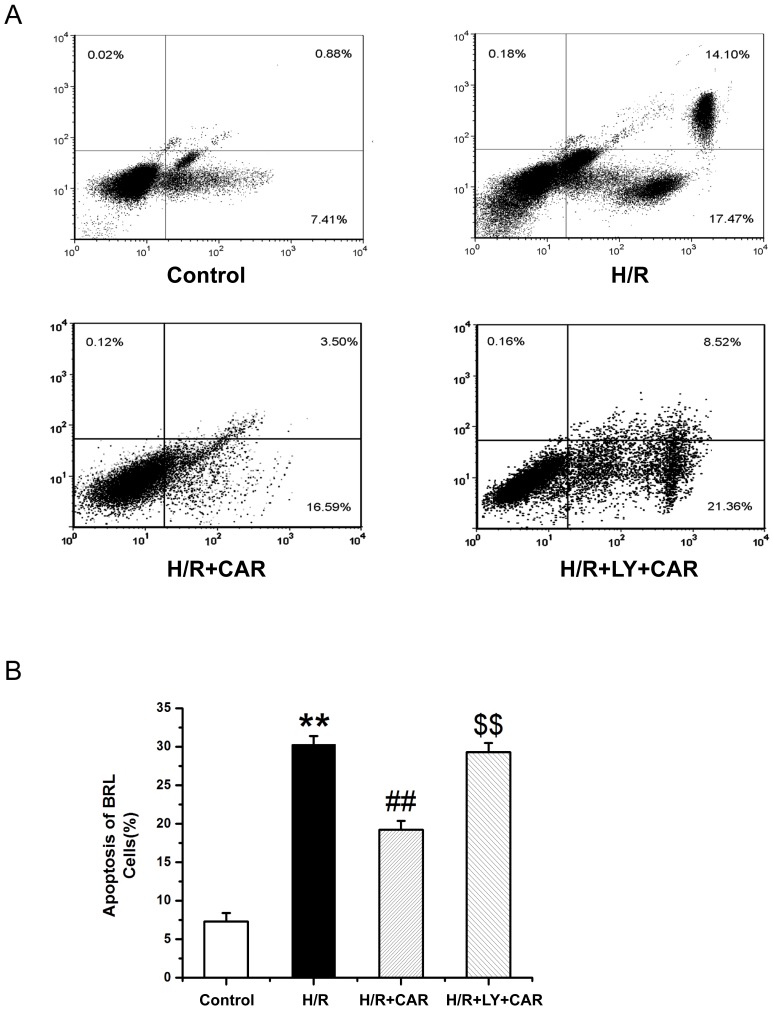
CAR inhibits apoptosis of H/R BRL cells *in vitro*. Untreated BRL cells (control), and BRL cells subjected to hypoxia (H/R), or subjected to hypoxia and treated with CAR (H/R+CAR), or subjected to hypoxia and treated with CAR+LY (H/R+LY+CAR) were analyzed for apoptosis by flow cytometry. Values were expressed as mean ± SEM (n = 8). ^**^ *P*<0.001 compared to the control group; ^##^ *P*<0.001 compared to the H/R group; ^$$^ *P*<0.001 compared to the H/R+CAR group.

**Figure 7:**
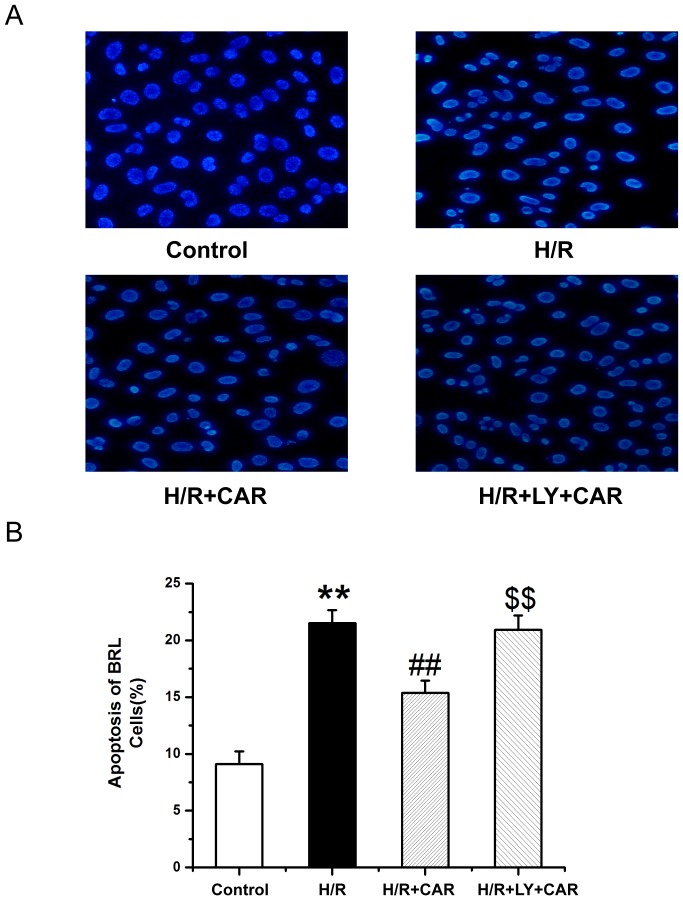
CAR inhibits apoptosis of H/R BRL cells *in vitro*. The cells in Figure 6 were further stained by Hoechst 33258. (B) Apoptotic cells were counted. Values were expressed as mean ± SEM (n = 8). ^**^ *P*<0.001 compared to the control group; ^##^ *P*<0.001 compared to the H/R group; ^$$^ *P*<0.001 compared to the H/R+CAR group.

Fig. 6 shows that BRL cells underwent significant apoptosis when exposed to H/R compared with untreated cells as analyzed by flow cytometry. Treatment with CAR significantly decreased the rate of apoptosis, compared with the untreated H/R cells However, pretreatment with LY294002 increased the apoptotic rate of H/R cells treated with CAR (*p*<0.001). Fig. 7 shows that in Hoechst 33258-stained BRL cells underwent significant apoptosis when exposed to H/R compared with untreated cells. Treatment with CAR significantly decreased the rate of apoptosis, compared with the untreated H/R cells Pretreatment with LY294002 significantly increased the apoptotic rate of H/R cells treated with CAR (*p*<0.001).

### CAR regulates apoptosis-related proteins

**Figure 8:**
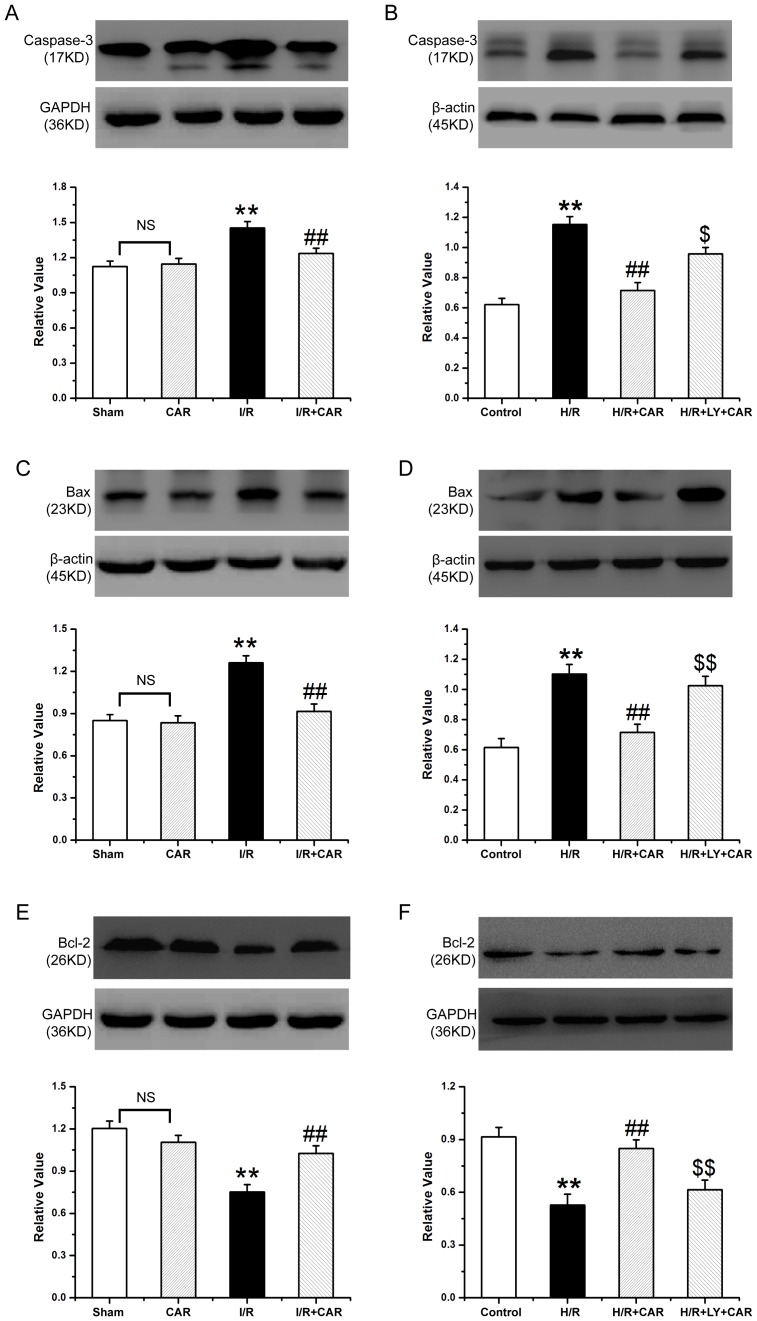
Detection of Caspase-3, Bax and Bcl-2 protein expression by western blot analysis. (A, C, E) The livers from the sham, CAR, I/R and I/R+CAR groups were collected. (B, D, F) The untreated BRL cells (control), and BRL cells subjected to hypoxia (H/R), or subjected to hypoxia and treated with CAR (H/R+CAR), or subjected to hypoxia and treated with CAR+LY (H/R+LY+CAR) were harvested. The tissues and cells were lysed and subjected to Western blot analysis to detect the expression of Caspase-3 (A, B), Bax (C, D), and Bax (E, F). Band density was measured and normalized to that of GAPDH. Data were expressed by mean ± SEM (n = 8). NS, no significant difference. ^**^ *P*<0.001 compared to the sham group or control cells; ^##^ *P*<0.001 compared to the I/R or H/R group. ^$^ *P*<0.05 and ^$$^ *P*<0.001 compared to the H/R+CAR group.

The expression level of Bcl-2 protein was decreased in livers from rats that underwent I/R compared to the sham group. The Bcl-2 expression was elevated in livers of rats in the I/R+CAR group, compared to the I/R group, and was no significantly different from that in the sham group. The opposite trend was found regarding the expression of Bax and Caspase-3 in rat livers, compared with Bcl-2 (*p*<0.05) (Fig. 8A). BRL cells expressed lower levels of Bcl-2 protein when exposed to H/R compared to untreated controls. The Bcl-2 protein level was upregulated in CAR-treated cells exposed to H/R, but addition of LY decreased its expression. The trend of Bax and Caspase-3 expression in BRL cells was similar to that in the rat liver tissues (*p*<0.05) (Fig. 8B).

### CAR enhances phosphorylation of Akt

**Figure 9:**
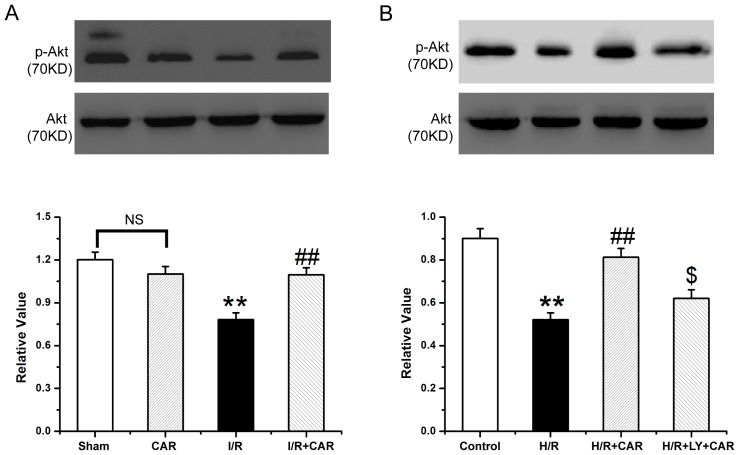
Detection of Akt and p-Akt expressions by western blot analysis. The lysates of liver tissues (A) and BRL cells (B) from Figure 8 were further Western blotted to detect the expression of p-Akt and total Akt. Band density was measured and normalized to that of total Akt. Data were expressed by mean ± SEM (n = 8). NS, no significant difference. ^**^ *P*<0.001 compared to the sham group or control cells; ^##^ *P*<0.001 compared to the I/R group or H/R group; ^$^ *P*<0.05 compared to the H/R+CAR group.

The expression level of p-Akt protein was decreased in livers from rats that underwent I/R, and was increased in the I/R+CAR group compared to the I/R group, and was not significantly different from that in the sham group, while the total Akt remained unchanged (*p*<0.05) (Fig. 9A). Similarly H/R BRL cells expressed lower levels of p-Akt protein i compared to untreated controls. The p-Akt protein level was up-regulated in the H/R+CAR cells, and addition of LY reduced its expression (*p*<0.05) (Fig. 9B). The total Akt had no significantly changes in BRL cells similar to that in rat liver tissues.

## Discussion

The present study has for the first time demonstrated that CAR protected against I/R injury in both rat liver and BRL cells, and the PI3K-Akt signaling pathway was involved in the protective mechanisms of CAR on I/R injury. Large numbers of studies have shown that CAR has bactericidal activity, fungicidal activity and insecticidal activity as a food additive. Furthermore, CAR has anti-depression activity and anti-tumorigenic activity in vitro and in vivo [27], [28].

IR is a physiopathologic process that mediates damage in several clinical settings. Several relevant factors have been involved in I/R injury, such as the accumulation of ROS, infiltration of neutrophilic granulocyte and apoptosis [29], [30]. Cumulative evidence indicates that ROS has the ability to increase the damage caused by I/R. The SOD and CAT level increased in the I/R injury of transgenic mice, and animals receiving injection of EC-SOD also had maintained GSH levels and reversed the increased liver MDA levels in comparison with those control groups [31]. It has been reported that CAR decreased the levels of ROS, increased the activity of Mn-SOD and CAT, and attenuated myocardial oxidative stress and apoptosis following myocardial ischemia-reperfusion in mice [32]. In the present study, we revealed that administration of CAR increased the levels of SOD, CAT and GSH, but decreased the level of MDA in the I/R rats, indicating that that CAR may alleviate I/R injury by preserving the SOD, CAT and GSH levels and reversing the increased MDA level. Canbek M et al firstly reported the protective effect of CAR against liver I/R in rats [18]. The present study has demonstrated similar results on the levels of AST, ALT and GSH, and hepatic histology, compared to that study [18]. In addition, we have further shown that CAR could inhibit apoptosis of liver cells *in vivo* and Buffalo rat liver cells *in vitro*, and conducted an investigation on the molecular mechanism accounting for the protective role of CAR against I/R.

Apoptosis can be induced through the mitochondrial, the death receptor and the endoplasmic reticulum stress pathways. The mitochondrial pathway is a classic pathway, during which apoptosis begins with the permeabilisation of the mitochondrial outer membrane [33]. Bcl-2 family members are divided into anti-apoptotic proteins such as Bcl-2 and pro-apoptotic proteins such as Bax [34], [35]. The main function of Bcl-2 is to stabilize mitochondrial membrane potential and inhibit Cyt-c release and caspase activation [36], [37]. However, Bax plays an opposite role compared with Bcl-2. The present study showed that the caspase-3 activity in rats treated with CAR was decreased compared with the sham group; in accompanied with decreased expression of Bax protein and increased expression of Bcl-2. Therefore, the results showed that CAR alleviated I/R injury by suppressing apoptosis.

The PI3K/Akt pathway is a critical survival mediator in the signal transduction pathways after ischemia, and is also essential for the regulation of proliferation, differentiation, and apoptosis [38]. Previous studies showed that the downstream factor of PI3K-Akt signal transduction pathwayis activated in I/R injury. Akt, the serine-threonine kinase, is a vital molecule in PI3K-Akt pathway and controls survival and apoptosis [39], [40] and suppresses apoptosis after being phosphorylated. It was reported that the expression level of p-Akt significantly increased in the initial stage of focal cerebral ischemia and decreased after reperfusion, while the expression of Akt was not changed [41]. Upregulation of p-Akt was induced by I/R injury [42]. In order to confirm the role of PI3K-Akt pathway in I/R injury, the present study focused on the expression of Akt and p-Akt. In accord, CAR increased the level of p-Akt and provided neuroprotection on focal cerebral I/R injury in mice [43]. Our results showed that treatment with CAR enhanced p-Akt compared to I/R group, while total Akt was not significantly changed. LY294002 as the special inhibitor of PI3K had striking effects on CAR treatment that it in part blocked p-Akt expression. Meanwhile, the findings showed that CAR could reduce I/R-induced liver injury by strengthening the anti-oxidant and anti-apoptotic activity. All the results suggested that CAR alleviates I/R injury by upregulating the expression of bcl-2 and p-Akt protein and downregulating the expression of Caspase-3 and Bcl-2 proteins.
